# Do Smarter People Have More Conservative Economic Attitudes? Assessing the Relationship Between Cognitive Ability and Economic Ideology

**DOI:** 10.1177/01461672211046808

**Published:** 2021-09-22

**Authors:** Alexander Jedinger, Axel M. Burger

**Affiliations:** 1 GESIS—Leibniz Institute for the Social Sciences, Cologne, Germany

**Keywords:** cognitive ability, intelligence, economic attitudes, economic ideology, meta-analysis

## Abstract

Evidence on the association of cognitive ability with economic attitudes is mixed. We conducted a meta-analysis (*k* = 20, *N* = 46,426) to examine the relationship between objective measures of cognitive ability and economic ideology and analyzed survey data (*N* = 3,375) to test theoretical explanations for the association. The meta-analysis provided evidence for a small positive association with a weighted mean effect size of *r* = .07 (95% CI = [0.02, 0.12]), suggesting that higher cognitive ability is associated with conservative views on economic issues, but effect sizes were extremely heterogeneous. Tests using representative survey data provided support for both a positive association of cognitive ability with economic conservatism that is mediated through income as well as for a negative association that is mediated through a higher need for certainty. Hence, multiple causal mechanisms with countervailing effects might explain the low overall association of cognitive ability with economic political attitudes.

In the political arena, actors often describe their opponents as incompetent or stupid (e.g., Anson, 2018; Mark, 2006). Indeed, empirical evidence supports the view that a link between cognitive abilities and political attitudes exists (e.g., Kanazawa, 2010; Meisenberg, 2015). More specifically, most studies indicate that lower cognitive abilities are linked to the endorsement of conservative political views (for overviews, see Onraet et al., 2015; Van Hiel et al., 2010). However, a closer inspection of the evidence on the ideology-ability link reveals that the association between lower scores in cognitive ability tests and conservative political preferences holds in particular for sociocultural attitudes (Onraet et al., 2015) while the evidence with respect to economic attitudes is much more inconsistent. In fact, some studies indicate that the correlation between cognitive abilities and economic conservatism tends to be positive rather than negative (e.g., Caplan & Miller, 2010; Carl, 2014; Johnston, 2018).

In the present research, we aimed at synthesizing the available empirical evidence on the link between cognitive abilities and economic ideology in a meta-analysis (Study 1) and at testing different theoretical accounts of the association using representative survey data (Study 2). In the following, we start with a short discussion of multidimensional models of political ideology and then give an overview of empirical findings on the association of cognitive abilities with (a) sociocultural attitudes and (b) economic attitudes. Next, we compare different theoretical perspectives on the link between cognitive abilities and economic attitudes before turning to our own analyses.

## Conceptualizing Political Ideology and Cognitive Abilities

Political ideology has been defined “as an interrelated set of attitudes and values about the proper goals of society and how they should be achieved” (Tedin, 1987 p. 65). A pertinent debate in political psychology concerns the extent to which political attitudes are organized along one or more ideological dimensions (for an overview, see Jost et al., 2009). According to one-dimensional accounts (e.g., Jost, 2006), most political conflicts in Western societies can be parsimoniously characterized as reflections of a single underlying left-right or liberalism-conservatism ideology dimension. In contrast to this view, multi-dimensional accounts of the structure of political attitudes usually argue in favor of distinguishing between at least two key dimensions of political ideology (Carmines & D’Amico, 2015; Duckitt, 2001; Eysenck, 1954; Jost et al., 2003; Lipset, 1960): The *sociocultural dimension* refers to the tension between personal freedom, autonomy, and diversity on the left and the maintenance of common identity and traditional social norms and values on the right. The left pole is also referred to as social liberalism and the right pole as social conservatism. In contrast, the *economic dimension* is characterized by a conflict between a preference for equality and the acceptance of regulatory interventions into the market on the left and a preference for self-responsibility and competition as well as acceptance of social inequality on the right. The two end poles have also been denoted as economic liberalism versus economic conservatism.^1^

The distinction between the sociocultural and the economic dimension of ideology refers to fundamental lines of tension concerning the proper way of organizing society. Specific political attitudes can be associated with both ideology dimensions (e.g., Jedinger & Burger, 2019, 2020). However, specific attitudes that are typically considered sociocultural (e.g., on immigration, women’s rights, or acceptance of homosexuality) are usually more strongly associated with the sociocultural ideology dimension while specific economic attitudes (e.g., on minimum wages, social welfare, or market optimism) are usually more strongly associated with the economic ideology dimension. Hence, specific political attitudes can be used as proxies for estimating a person’s ideological orientation (e.g., Everett, 2013).

The distinction between the ideology dimensions is relevant because empirical evidence shows that sociocultural ideology and economic ideology represent empirically distinct dimensions of ideological thinking that are rooted in different psychological dispositions (e.g., Duckitt & Sibley, 2010; Feldman & Johnston, 2014; Johnston & Ollerenshaw, 2020; Middendorp, 1978; Treier & Hillygus, 2009; Zumbrunnen & Gangl, 2008). Hence, the ideological dimensions do not necessarily have to be congruent in the sense of a classical left-right schema with culturally and economically conservative attitudes on the right and culturally and economically liberal positions on the left. For example, recent research demonstrates that a left-authoritarian attitude structure where cultural conservative views are combined with left-wing economic positions is not uncommon in mass publics (Lefkofridi et al., 2014; Malka et al., 2019).

When it comes to the association of ideological orientations with psychological dispositions and—more specifically—with cognitive abilities, Adorno and his colleagues (1950) were among the first to propose that lower intelligence and rigid styles of information processing are related to conservative social and economic attitudes. The terms *cognitive ability* and *intelligence* refer to “a highly general information-processing capacity that facilitates reasoning, problem solving, decision making, and other higher order thinking skills” (Gottfredson, 1997, p. 81). Scores on measures of specific cognitive abilities such as verbal ability, quantitative ability, spatial ability, or abstract reasoning are positively correlated, which has been explained by the proposition of an underlying general intelligence factor, commonly known as Spearman’s *g* (Gottfredson, 1997; Johnson et al., 2004; Spearman, 1904 but see Van Der Maas et al., 2006). Other researchers (e.g., Thurstone, 1938) have argued in favor of distinct factors of intelligence instead of a single general factor. Hierarchical models of intelligence reconcile both views by postulating a general intelligence factor on the most abstract level of a hierarchy as well as subfactors on different lower levels of abstraction (Carroll, 1993; Cattell, 1971; Horn & Noll, 1997).

Cognitive ability and intelligence are conceptually and empirically distinct from *cognitive style* (Stanovich, 2012), which refers to individual differences in preferred and habitual modes of information search and information processing, such as holistic versus analytic thinking (e.g., Talhelm et al., 2014) or intuitive versus reflective judgment and decision making (e.g., Epstein et al., 1996). As pointed out by Onraet et al. (2015), individual differences in cognitive style have received more attention in research on the psychological bases of political attitudes than individual differences in cognitive abilities. Despite the stronger focus of research on cognitive style, the investigation of the role of cognitive abilities in social and political attitudes has flourished in recent years.

## Cognitive Abilities and Sociocultural Attitudes

Currently, a large body of work indicates a negative association between measures of cognitive ability and the endorsement of conservative sociocultural attitudes (Onraet et al., 2015; Schoon et al., 2010; Van Hiel et al., 2010). For example, higher scores in right-wing authoritarianism (RWA) have been shown to be associated with lower scores in cognitive tasks (Burger et al., 2020; Choma et al., 2019; De keersmaecker et al., 2018; Heaven et al., 2011). In a large-scale, nationally representative UK sample, lower general intelligence in childhood has been found to predict the endorsement of conservative ideology at an adult age when controlling for education and socioeconomic status (Hodson & Busseri, 2012). With respect to voting behavior, lower cognitive abilities were associated with more intentions to vote for Donald Trump and less intentions to vote for Hillary Clinton in the 2016 US presidential elections through effects on right-wing authoritarianism and social dominance orientation (Choma & Hanoch, 2017; Ganzach et al., 2019). Even temporary reductions in cognitive resources through alcohol intoxication, cognitive load, or time pressure have been argued to facilitate the endorsement of conservative views (Eidelman et al., 2012). In a meta-analysis by Onraet and colleagues (2015) that included 67 studies, 57 studies showed negative relations, nine studies showed positive relations, and one study showed no relation between cognitive abilities and conservative attitudes. The average effect size was *r* = −.20, which supports the view that a link between cognitive abilities and political views exists but shows also that individual differences in cognitive ability are only moderately related to political views.

## Cognitive Abilities and Economic Attitudes

In research on the association of cognitive abilities with political attitudes, economic attitudes have received less attention than sociocultural attitudes, so far. Although the role of cognitive abilities in the formation of economic attitudes has become more prominent in recent years, empirical results are inconsistent. Several studies provide evidence for a positive association of cognitive abilities with economic conservatism: Using data from the General Social Survey (GSS), some studies have shown that verbal intelligence is associated with opposition to governmental regulation of markets and the redistribution of income (Caplan & Miller, 2010; Carl, 2014; Ganzach, 2018, Study 1; Kanazawa, 2010, Study 2). Similarly, in an analysis of data from the 2012 American National Election Study (ANES), Carl (2015) demonstrated that individuals with higher verbal intelligence are more likely to embrace fiscally conservative beliefs. Outside the United States, Oskarsson et al. (2015) observed that higher levels of intelligence were related to preferences for privatization, lower taxes, and less redistribution of wealth among Swedish male twin pairs. Ludeke and Rasmussen (2018, Study 2) matched ability test scores from Danish draftees with survey data on economic attitudes and found a positive relationship between intelligence and economic laissez-faire orientations (see also Rasmussen, 2016).

While most of these results are robust to using demographic controls such as educational attainment, Johnston (2018) found no relationship between verbal intelligence and economic policy opinions in US data once the level of education was controlled for. Other researchers specifically explored the role of education and social status as possible mechanisms underlying the link between intelligence and economic conservatism: In Sweden, Mollerstrom and Seim (2014) combined survey responses with intelligence test scores from military enlistment records. Their results indicate that higher cognitive abilities were associated with demanding less income redistribution among Swedish males and that this association was partially accounted for by a higher annual income and the belief that economic success is based on individual effort rather than luck (see also Karadja et al., 2017). Using data from two longitudinal cohort studies in the United Kingdom, Lewis and Bates (2018) reported that higher levels of intelligence in childhood were associated with more economically conservative attitudes in adulthood. Interestingly, they found support for the hypothesis that higher socioeconomic status (educational attainment and social class) in adulthood mediates part of the effect of childhood intelligence on adult economic attitudes.

Other studies failed to find evidence for a relationship between cognitive abilities and economic attitudes at all, both using single-item economic orientation questions (Choma et al., 2019; Pennycook et al., 2014) as well as longer batteries of economic issue opinions (Kirkegaard et al., 2017; Saribay & Yilmaz, 2017; Yilmaz & Saribay, 2016, Study 2). Finally, Sterling et al. (2016) found that higher performance on several cognitive ability measures was associated with less endorsement of free-market positions and self-identified economic liberalism in the US-American sense. Taken together, the empirical findings presented so far provide mixed evidence about the association of cognitive abilities with economic attitudes.

## Explaining the Link Between Cognitive Ability and Economic Ideology

As a step toward understanding the inconsistency of findings on the association of cognitive abilities with economic attitudes, it is worthwhile to consider the theoretical explanations for such a link that have been put forward in the literature. In the following, we distinguish between three theoretical accounts.

### The Self-Interest Hypothesis

A straightforward explanation for a positive association of cognitive ability scores with economic conservatism parts from the idea that higher cognitive abilities are associated with higher levels of formal education (possibly, with causal links in both directions). Higher formal education, in turn, constitutes and facilitates higher social and economic status. As a consequence, according to this view, high-status individuals have more to lose from governmental redistribution of their (anticipated) wealth than low-status individuals (Johnston, 2018). Hence, they are less supportive of governmental regulations of markets, progressive taxation, and social welfare policies due to self-interest.

As discussed above, a range of empirical findings are consistent with the self-interest hypothesis by showing that indicators of socioeconomic status account for the association of cognitive abilities with ideological orientations fully (Lewis & Bates, 2018; Mollerstrom & Seim, 2014) or at least partially (Caplan & Miller, 2010; Carl, 2014; Ganzach, 2018, Study 1; Kanazawa, 2010, Study 2). In a recent study, Ganzach (2020) demonstrated that education and income—two components that are typically treated as indicators of socioeconomic status—differentially mediated the effect of intelligence on political ideology: Cognitive ability fostered liberal views via its positive effect on education, whereas higher levels of cognitive ability were positively related to income which in turn fueled conservative political orientations.

### The Economic Sophistication Hypothesis

According to this view, understanding the rationale and benefits of typically conservative economic policy principles such as free markets, comparative advantages, economic competition, privatization, and restrictive welfare programs requires more background knowledge than understanding the rationale of left-wing economic policy principles such as the redistribution of wealth and strong social support systems. In other words, more intelligent people “think more like economists” (Caplan & Miller, 2010, p. 636). Lay economic thinking is associated with a number of cognitive biases, such as the assumption of a fixed pie of societal resources that can be redistributed or the do-no-harm heuristic that leads to the rejection of economic measures that harm a small group of people but make society as a whole better off (Baron et al., 2006; Caplan, 2007). According to this view, people of lower intelligence are more prone to lay economic thinking and therefore develop a desire for more government intervention and tend to be more skeptical about pro-market policies than individuals with higher cognitive abilities (Caplan & Miller, 2010).

### The Epistemic Needs Hypothesis

In contrast to the previous accounts, the ideology-as-motivated-cognition perspective (Jost et al., 2003) entails the prediction of a negative association between cognitive abilities and economic conservatism. At the core of this approach lies the assumption that chronically or temporarily increased existential and epistemic needs facilitate the endorsement of conservative positions on the sociocultural as well as the economic dimension. More specifically, two core elements of conservatism within many Western societies—resistance to change and acceptance of inequality—are seen as instrumental for satisfying existential and epistemic needs by providing protection from potentially destabilizing reforms toward more sociocultural pluralism and more economic and social equality.^2^ We refer to this view—also termed the rigidity-of-the-right hypothesis (e.g., Malka & Soto, 2015; Tetlock, 1984)—as the epistemic needs hypothesis because we consider epistemic needs at the core of the mechanism that links cognitive abilities to economic political attitudes. Even though the ideology-as-motivated-cognition perspective usually refers to measures of epistemic needs and cognitive style, it might also explain the link between cognitive abilities and political attitudes given that lower cognitive abilities have been shown to be linked to stronger epistemic needs such as the need for cognitive closure (De keersmaecker et al., 2018). In fact, some explanations offered for the link between cognitive abilities and political attitudes fit the ideology-as-motivated-cognition perspective and its epistemic needs hypothesis very well. For example, Onraet et al. (2015) hypothesize that “those with fewer cognitive resources drift towards right-wing conservative ideologies in an attempt to increase psychological control over their context” (p. 601).

## The Present Research

In the present research, we first integrated the extant empirical evidence on the association of cognitive ability with economic attitudes in a meta-analysis (Study 1) and then tested hypotheses derived from different theoretical explanations for this association in an analysis of U.S. survey data (Study 2). We extended previous research on the link between cognitive ability and political attitudes (e.g., Onraet et al., 2015) by focusing on economic attitudes and by putting some of the most important explanations for this link to an empirical test. In both studies, we focused specifically on economic ideologies and attitudes rather than superordinate generalized attitudes such as right-wing authoritarianism (Altemeyer, 1981) or social dominance orientation (Pratto et al., 1994). In addition, our analyses are restricted to objective measures of cognitive abilities rather than self-reports or interviewer assessments.

## Study 1

### Method

#### Selection of studies

To identify relevant empirical studies for our meta-analysis, we searched various online databases (e.g., ISI Web of Knowledge, PsycINFO)^3^ using a combination of the following keywords: *economic attitudes*, *economic beliefs*, *economic ideology*, *economic conservatism*, *economic liberalism*, *cognitive ability*, *mental ability*, *intelligence*, *IQ*, and *wordsum*.^4^ In addition, we employed a backward and forward citation search to locate additional studies that were not indexed by the aforementioned literature databases.

To be included in our meta-analysis, studies had to meet the following criteria. First, the study had to include at least one behavioral measure of cognitive ability. That is, studies that used self-report measures of cognitive ability or intelligence were not taken into account, as well as studies that employed measures of cognitive style such as variants of the cognitive reflection test (CRT; Frederick, 2005) and measures of cognitive rigidity (e.g., Zmigrod et al., 2019). Second, the study examines at least one aspect of economic ideology. Economic ideology is defined as beliefs about how the economy does or should work. This encompasses views on how the production, exchange, distribution and consumption of goods and services are organized and the role of government in these processes, as well as attitudes toward the redistribution of economic resources. Thus, we excluded studies that employed generalized ideological attitudes such as social dominance orientation, justice ideologies or (anti-)egalitarianism. Third, we focused on quantitative studies that contain sufficient statistical information to calculate zero-order correlations as measures of effect size. If this information was not available, the authors were contacted to provide the necessary statistics or the corresponding raw data with up to three contact attempts over a 3-week period.

**Figure 1.**
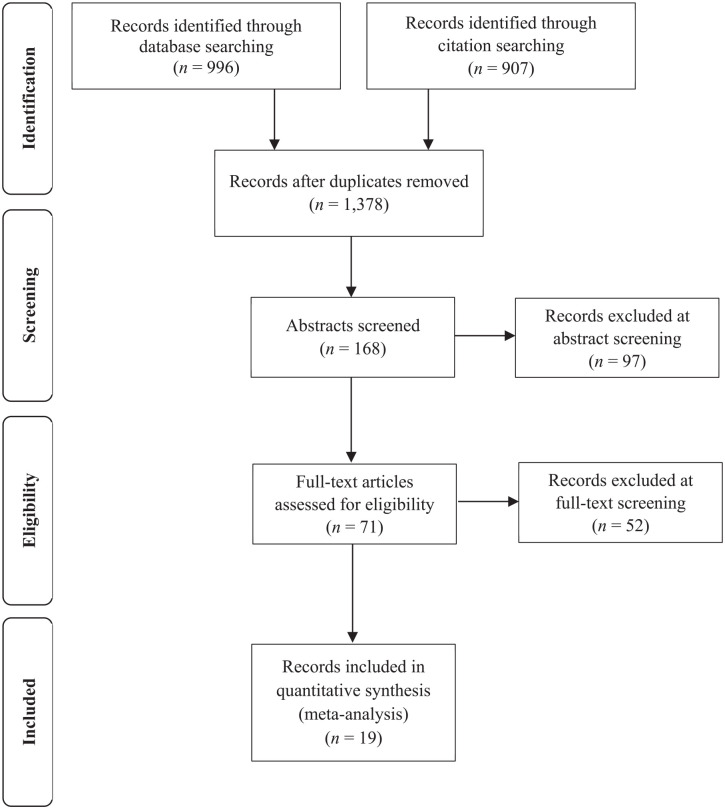
PRISMA flowchart of study selection.

Figure 1 shows a flowchart for the selection of studies. After duplicates were removed, our search resulted in 168 records whose abstracts were screened. We excluded 97 articles that did not actually investigate the association of cognitive abilities with economic attitudes. Out of the remaining 71 articles, 52 were not suitable for the present meta-analysis because they were theoretical or review articles, did not contain any measures of economic ideology, or no effect sizes were reported, and authors did not respond to queries. Furthermore, four studies were excluded because they relied on exactly the same data as earlier studies from the same authors (Carl, 2015; Karadja et al., 2017; Lewis, 2018; Ludeke & Rasmussen, 2018).

Finally, we identified 19 records that were, in principle, eligible for inclusion in the present meta-analyses. However, during the coding of the studies, we found that four studies used data from different but overlapping waves of the GSS (Caplan & Miller, 2010; Carl, 2014; Ganzach, 2018 Study 1; Kanazawa, 2010, Study 2). The GSS is a nationally representative multitopic survey that has been measuring the verbal intelligence of respondents with the so-called Wordsum test since 1974 (Cor et al., 2012). To maintain the statistical independence of the effect sizes from these four studies, we accessed the original data and reanalyzed the reported correlations following the original study protocols as closely as possible. This enabled us to exclude redundant associations and to include the four studies as a joint study in our analysis. As seen in Table 1, the final sample included 19 articles with 23 studies and *k* = 20 independent effect sizes (*N* = 46,426).

**Table 1.** Summary of Studies Included in the Meta-Analysis.

Study ID	Author(s)	Effect size (*r*)	Total *N*	Population	Data source	Cognitive ability measure(s)	Economic ideology measure	No. of items in ideology measure(s)
1	Caplan & Miller (2010)	.06	2,373	American adults	GSS	Wordsum	Operational	17
2	Carl (2014)	.21	1,247	American adults	GSS	Wordsum	Operational	6
3	Carl (2015)	.12	5,394	American adults	ANES 2012	Wordsum	Operational	24
4	Choma et al. (2019)—Study 1	−.06	455	American adults	MTurk	Objective Numeracy Scale	Symbolic	1
5	Choma et al. (2019)—Study 2	−.06	406	American adults	MTurk	Objective Numeracy Scale	Symbolic	1
6	Cor et al. (2012)	.21	3,519	American adults	Mixed	Wordsum	Operational	1
7	Ganzach (2018)—Study 1	.16	4,515	American adults	GSS	Wordsum	Operational	3
8	Ganzach (2018)—Study 2	−.03	3,841	American adults	NLSYC	PPVT	Operational	1
	GSS^a^	.10	2,373	American adults	GSS	Wordsum	Operational	30
9	Johnston (2018)	.02	3,396	American adults	ANES 2016	Wordsum	Operational	14
10	Kanazawa (2010)—Study 2	.20	5,827	American adults	GSS	Wordsum	Operational	2
11	Kemmelmeier (2008)—Study 1	−.02	4,901	American students	CIRP	SAT, ACT	Operational	1
12	Kirkegaard et al. (2017)	.07	259	Danish adults	Online Panel	ICAR-5	Operational	10
13	Lewis & Bates (2018)—BCS 1970	.19	6,736	British children/adults	BCS70	British ability scales	Operational	6
14	Lewis & Bates (2018)—NCDS 1958	.25	8,961	British children/adults	NCDS58	General ability test	Operational	6
15	Mollerstrom & Seim (2014)	.25	271	Swedish adult men	Statistics Sweden	Swedish military test	Operational	1
16	Nilsson et al. (2019)	.10	985	Swedish adults	Online Panel	Numeracy	Symbolic	1
17	Oskarsson et al. (2015)	.21	1,946	Swedish adult men	SALTY	SALTY, Swedish military test	Operational	8
18	Pennycook et al. (2014)	−.05	505	American adults	MTurk	Numeracy, Wordsum	Symbolic	1
19	Rasmussen (2016)—Study 1	.00	948	Danish adults	Danish Draftee Sample	BPP	Operational	3
20	Rasmussen (2016)—Study 2	.06	1,408	American adults	MTurk	ICAR	Operational	3
21	Saribay & Yilmaz (2017)	−.05	376	American adults	MTurk	Wordsum, Base-rate neutral problems	Mixed	6
22	Sterling et al. (2016)	−.13	163	American adults	MTurk	Wordsum, Numeracy, RAPM	Mixed	6
23	Yilmaz & Saribay (2016)—Study 2	.03	403	Turkish students	Psychology course	Cognitive ability test	Operational	16

*Note.* GSS = General Social Survey; ANES = American National Election Study; MTurk = Amazon Mechanical Turk; NLSYC = National Longitudinal Study–Children; CIRP = Cooperative Institutional Research Program; BCS70 = British Cohort Study 1979; NCDS58 = National Child Development Study 1958; SALTY = Screening across the Life-span Twin [Younger] cohort study.

a To maintain the statistical independence of the studies that relied on data from the GSS (Caplan & Miller, 2010; Carl, 2014; Ganzach, 2018, Study 1; Kanazawa, 2010, Study 2), we reanalyzed the original data and included the four studies as a joint study in our meta-analysis.

#### Coding of study characteristics

Various study characteristics were coded to examine possible moderator variables. For each study, we coded which *measures of cognitive ability* were administered. The majority of studies relied on the Wordsum test (*n* = 7), an objective numeracy test (*n* = 3) or a mix of both instruments (*n* = 3). As described above, Wordsum is a short vocabulary test that asks respondents to identify one word in a set of five whose meaning is closest to a target word (Cor et al., 2012). Numeracy tests measure the ability to understand statistical information by performing mathematical operations involving proportions, percentages, and probabilities, and they are available in different formats (e.g., Lipkus et al., 2001; Schwartz et al., 1997).

The studies under investigation used a variety of instruments to *measure economic ideologies*. These included self-placement on an economic liberal-conservative or left-right continuum (*n* = 4) as well as scales that assessed specific positions related to economic policies (*n* = 17). Other studies combined ideological self-placement with policy attitudes (*n* = 2). Accordingly, we classified measures of economic ideology as symbolic, operational or mixed. This categorization is important because there is evidence that both symbolic and operational measures represent different aspects of ideological thinking (Conover & Feldman, 1981; Ellis & Stimson, 2012). Ideological self-identification reflects the affective attachment to political in-groups and their symbols and not necessarily a person’s attitudes toward specific political issues. Symbolic ideology is typically assessed by a single self-placement item (e.g., “How liberal or conservative do you tend to be when it comes to economic policy?”; Choma et al., 2019). However, terms like “economically conservative” or “economically liberal” are inherently vague and may be interpreted in very different ways by different respondents, especially when people have a poor understanding of economic concepts (see Bauer et al., 2017). In contrast, operational ideology focuses on preferences for concrete policy proposals (e.g., whether an individual should be more responsible for himself or the public sector should be responsible for taking care of all; Rasmussen, 2016). The two dimensions of ideological thinking do not necessarily have to be congruent. Research has shown, for example, that people who identify themselves as conservatives may support liberal policies such as social redistribution programs at the operational level and vice versa (e.g., Ellis & Stimson, 2012).

Unfortunately, no standard for the operational measurement of economic beliefs has yet been established. Although there are some psychometrically tested scales (e.g., Everett, 2013; Henningham, 1997), instruments to measure economic policy attitudes are usually formed ad hoc, which makes it difficult to compare the correlations obtained with cognitive abilities. While the internal reliability of these ad hoc scales is often quite high (e.g., Kirkegaard et al., 2017; Lewis & Bates, 2018; Rasmussen, 2016), their construct validity is rarely tested. Thus, the inconclusiveness of the results described in the literature review may also be due to the heterogeneity of the measurement of economic ideologies.

Although, we had no leverage to control for the validity of ideological measures, we coded the *number of items* of the scales assuming that more comprehensive scales produced more reliable estimates of effect size. Most studies employed between 2 and 10 items (*n* = 11), eight studies used a single item, and four studies employed more than ten items to measure economic ideology. Another factor that could influence the relationship between cognitive skills and economic orientations is the *sampling methodology*. Interestingly, most studies were based on representative population samples (*n* = 13), while a smaller number used self-selected (non-probability) samples to recruit subjects (*n* = 10), for example, from Amazon’s Mechanical Turk (MTurk). Finally, we extracted basic *study characteristics* relating to the time period and location of data collection (USA, *n* = 15; Scandinavia, *n* = 5; UK, *n* = 2; Turkey, *n* = 1).

#### Meta-analytic procedures and estimations of effect sizes

In the present meta-analysis, we used a random-effects (RE) model with a restricted maximum likelihood (REML) approach to estimate effect sizes. In contrast to fixed-effects models, RE models assume that the true effect sizes over studies follow a distribution whose mean value represents the average effect (Borenstein et al., 2010; Card, 2016). Thus, the models take into account that the observed heterogeneity of the estimated effects is due to not only natural fluctuations of the samples (sampling error) but also, possibly, other sources because the effect size estimates are not drawn from a single population. This allows us to generalize the results beyond the studies that we have included in our meta-analysis.

Pearson’s *r* was used to calculate the effect sizes. All measures of economic ideology were coded so that higher values indicate a higher degree of economic conservatism. For the present purpose, we define economic conservatism in the US-American sense as opposition toward governmental intervention in markets and the acceptance of economic inequality (see also Crowson, 2009; Zumbrunnen & Gangl, 2008). Thus, positive correlations indicate that higher levels of cognitive ability are associated with a more conservative economic ideology. If a study reports separate correlations for different measures of cognitive abilities and/or a set of economic issue positions, these effect sizes were transformed into Fisher-*z* values, averaged, and then transformed back into a correlation coefficient, which was then coded as the total effect size for the study. The data and code are openly available on the Open Science Framework at https://osf.io/36brp/.

### Results and Discussion

#### Overall effect of cognitive ability on economic ideology

**Figure 2.**
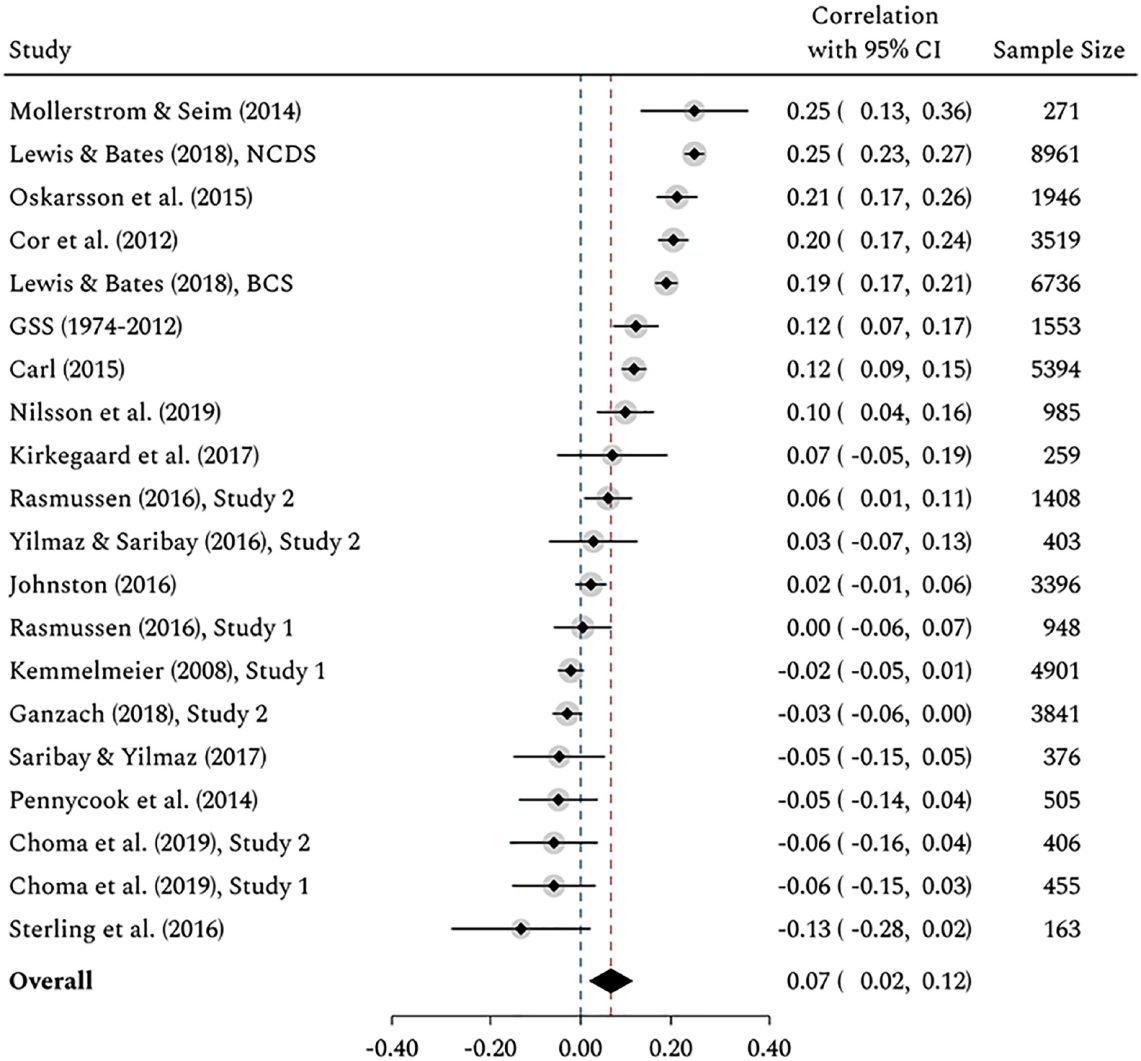
Forest plot of the effects of cognitive ability on economic ideology. *Note.* Positive effect sizes indicate that higher levels of cognitive ability are associated with a more conservative economic ideology. The diamond represents the overall estimated effect size and its 95% confidence interval using a random-effects model. Homogeneity: *Q*(19) = 551.43, *p* < .001, *I*^2^ = 96.2%. NCDS = National Child Development Study; BCS = British Cohort Study.

Overall, cognitive ability was positively and significantly associated with economic conservatism, *r* = .07, *z* = 2.67, *p* = .008, 95% CI = [0.02, 0.12] (see Figure 2).^5^ The magnitude of this weighted average correlation corresponds to a relatively small effect size (Gignac & Szodorai, 2016). However, the extent of heterogeneity among reported effect sizes is striking, τ^2^ (effect size variance) = 0.01, τ (effect size standard deviation) = 0.11, *Q*(19) = 551.43, *p* < .001. As indicated by the *I*^2^ statistic, 96.2% of the variability among effect sizes is caused by systematic factors and cannot be attributed to sampling errors alone. As a point of reference, 25%, 50% and 75% in terms of the variability of effect sizes correspond to low, moderate and substantial heterogeneity in meta-analyses (Higgins & Thompson, 2002). Another way to illustrate between-study heterogeneity is to use prediction intervals, which indicate the range of predicted effect sizes in a potential future study (Borenstein et al., 2017; Higgins et al., 2009).^6^ In the present case, we would predict that the correlations lie somewhere between −0.16 and 0.29 for 95% of similar studies that will be conducted in the future. This means that, although cognitive ability is on *average* positively associated with a more conservative economic outlook, the possible range of effect sizes contains negative associations. The high degree of heterogeneity among effect sizes suggests that the relationship between cognitive ability and economic ideology depends strongly on third variables.

#### Moderator analyses

To examine potential sources of heterogeneity among studies, we conducted a series of categorical moderator analyses. Following Onraet et al. (2015), we set the significance level to *p* < .01 (.05/5) to adjust for multiple comparisons. The results indicated that the type of cognitive ability measure (general, verbal, or numerical) did not significantly moderate the relationship between cognitive ability and economic ideology, *Q*(2) = 1.80, *p* = .41. We next tested whether effect sizes differed among the types of ideology measures administered, which yielded a significant difference, *Q*(2) = 14.34, *p* = .001. Cognitive abilities were positively associated with operational economic ideology (*r* = .11, *z* = 3.89, *p* = .0001, 95% CI = [0.05, 0.16]), while studies that assessed economic preferences with symbolic (*r* = −.01, *z* = −0.28, *p* = .78, 95% CI = [−0.10, 0.07]) or mixed scales (*r* = −.07, *z* = −1.70, *p* = .09, 95% CI = [−0.16, 0.01]) showed no significant associations.^7^

Contrary to our expectations, effect sizes did not significantly differ as a function of the number of items employed, *Q*(2) = 0.66, *p* = .72. However, the type of sampling methodology was a significant moderator of effect sizes, *Q*(1) = 11.89, *p* = .001. Cognitive ability was more strongly related to endorsements of free-market positions among probability samples of the population (*r* = .13, *z* = 4.05, *p* = .001, 95% CI = [0.07, 0.20]) than among self-selected samples of students or MTurk workers (*r* = −.002, *z* = −0.11, *p* = .92, 95% CI = [−0.05, 0.04]), which might be a consequence of higher homogeneity of the latter type of sample in terms of cognitive ability and/or political attitudes. Finally, the location of the study conditioned the magnitude of effect sizes, *Q*(1) = 7.34, *p* = .007, with stronger associations emerging in Scandinavian, British or Turkish samples (*r* = .14, *z* = 4.03, *p* = .0001, 95% CI = [0.07, 0.21]), while North American samples showed no significant associations (*r* = .02, *z* = 0.74, *p* = .46, 95% CI [−0.04, 0.08]) (Table 2).

**Table 2.** Moderators of the Effect of Cognitive Ability on Economic Ideology.

Moderator	*k*	*N*	*r*	95% CI	95% PI	Homogeneity tests		
						*Q*_between_	*Q*_within_	*I* ^2^
Overall effect	20	46,426	.07**	[0.02, 0.12]	[−0.16, 0.29]		551.43***	96.2%
Measures of cognitive ability						1.80		
General	12	26,877	.08*	[0.002, 0.15]	[−0.21, 0.34]		354.04***	96.6%
Verbal	5	17,703	.09*	[0.01, 0.17]	[−0.23, 0.39]		125.73***	96.7%
Numerical	3	1,846	−.001	[−0.11, 0.11]	[−0.87, 0.86]		11.76**	80.7%
Measure of economic ideology						14.34**		
Symbolic	4	2,351	−.01	[−0.10, 0.07]	[−0.36, 0.34]		14.02**	74.9%
Operational	14	43,536	.11***	[0.05, 0.16]	[−0.12, 0.32]		484.39***	96.6%
Mixed	2	539	−.07	[−0.16, 0.01]	—		0.82	0.00%
Number of items						0.66		
1	8	14,883	.04	[−0.04, 0.13]	[−0.26, 0.34]		165.46***	95.8%
2–10	8	20,797	.09	[−0.003, 0.18]	[−0.24, 0.39]		136.05***	97.0%
>10	4	10,746	.08**	[0.02, 0.13]	[−0.17, 0.32]		23.12***	85.1%
Sampling methodology						11.89**		
Non-probability	10	9,861	−.002	[−0.05, 0.04]	[−0.13, 0.13]		27.28**	68.6%
Probability	10	36,565	.13***	[0.07,0.20]	[−0.11, 0.37]		339.56***	97.3%
Location						7.34**		
USA	12	25,917	.02	[−0.04, 0.08]	[−0.19, 0.23]		198.95***	94.3%
Scandinavia/UK/Turkey	7	20,509	.14***	[0.07, 0.21]	[−0.10, 0.37]		90.48***	94.6%

*Note.* Positive effect sizes indicate that higher levels of cognitive ability are associated with a more conservative economic ideology. *k* = number of independent samples, CI = confidence interval, PI = prediction interval.

* *p* ≤ .05. **p < .01. ***p < .001.

#### Publication bias and sensitivity analyses

**Figure 3.**
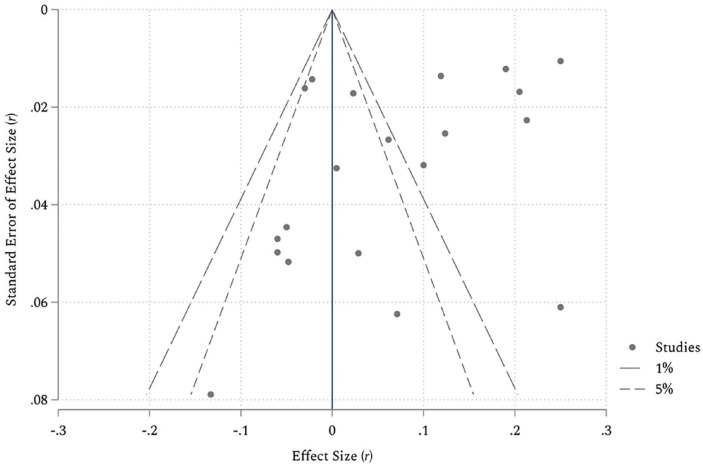
Contour-enhanced funnel plot of the effects of cognitive ability on economic ideology. *Note.* Dashed lines indicate the 5% and 1% significance contours, respectively.

A potential threat to the conclusions of meta-analyses is publication bias, sometimes also called small-study bias (Card, 2016). This means that studies with statistically nonsignificant results have less chance of being published, which distorts the estimated average effect size. This mostly concerns studies with smaller sample sizes and lower power to detect statistically significant results. Figure 3 shows a contour-enhanced funnel plot in which the observed effect sizes (*r*) are plotted against their precision (standard errors) accompanied by common regions of statistical significance (Peters et al., 2008). If there is no bias present, studies should be symmetrically distributed around the average effect size, with studies with larger effect sizes (more precise studies) at the top and studies with smaller effect sizes (less precise) at the bottom, which creates the appearance of an inverted funnel. A small-study bias is typically shown by the fact that studies are distributed asymmetrically around the average effect size and smaller (less precise) studies seem to be missing in the region of insignificance.

As seen in Figure 3, the distribution in our case is asymmetrical, but this mainly concerns large studies that concentrate on the right-hand side of the plot and thus suggest a positive association between intelligence and economic conservatism. The visual impression was confirmed by Egger’s test for asymmetry (Egger et al., 1997), which regressed the effect size on its standard error, *b* = −2.65, *SE* = 1.26, *z* = −2.10, *p* = .04. As we have seen, however, our sample of studies is characterized by extreme heterogeneity, which can also cause an asymmetric distribution of effect sizes (Sterne et al., 2011). In other words, the asymmetry is mainly due to factors like the type of ideological measure or sampling methodology and cannot be attributed to a publication bias based on statistical significance. This is because the area where studies seem to be “missing” (on the left-hand side of the plot) covers significant negative associations between cognitive ability and economic ideology, which are unlikely to disappear in file-drawers (Peters et al., 2008). To account for the between-study heterogeneity, we repeated Egger’s test with the significant moderators as covariates. The results yielded no significant evidence for funnel plot asymmetry, *b* = −1.11, *SE* = 1.49, *z* = −0.75, *p* = .46, which lends further support to the heterogeneity interpretation of the funnel plot asymmetry.

## Study 2

The meta-analytic results of Study 1 indicate a weak average association between cognitive ability and economic ideology as well as strong heterogeneity of the size and sign of the observed associations. At the same time, the theoretical explanations for the link between mental abilities and economic attitudes are also heterogeneous with respect to the sign of the association and the mediating mechanism they propose. While Study 1 focused on synthesizing the empirical evidence on the association of cognitive ability with economic attitudes as such, Study 2 sought to investigate the mechanisms underlying this association by systematically testing hypotheses derived from different theoretical perspectives: First, the *self-interest hypothesis* posits that the link between cognitive ability and economic ideology is accounted for by socioeconomic status. Individuals with higher cognitive skills are more likely to attain a higher socioeconomic status which compels them to adopt more conservative views on economic issues. Second, the *economic sophistication hypothesis* posits that cognitive ability is positively associated with economic knowledge, which in turn predicts a more conservative economic outlook. Third, the *epistemic needs hypothesis* suggests that epistemic needs mediate between cognitive ability and economic ideology. Specifically, cognitive ability is negatively associated with needs for security and certainty, which in turn are positively associated with economic conservatism. This mechanism should facilitate a negative overall relationship between cognitive ability and economic conservatism. Importantly, the different explanations are not mutually exclusive and countervailing effects of the different mechanisms could explain the low overall correlation observed in Study 1.

An a priori Monte Carlo power analysis (Schoemann et al., 2017) indicated that we need a simple size of at least *N* = 1,300 to detect a small indirect effect (.01) with adequate power (90%). Therefore, we relied on the 2016 ANES, which is a continuing program of high-quality surveys of sufficient sample size and contains several questions regarding attitudes toward economic issues. Moreover, the 2016 ANES included the established Wordsum test as a proxy for cognitive ability. The data and materials are openly available on the ANES website at https://electionstudies.org.

### Method

#### Participants

The 2016 ANES was a two-wave panel survey, administered face-to-face and online on a probability sample of U.S. citizens aged 18 or older (for methodological details, see DeBell et al., 2018). The sample included *N* = 4,271 pre-election interviews and *N* = 3,649 post-election re-interviews. The following analyses are limited to participants who took part in the pre- and post-election waves and provided complete answers to all variables under study, leaving a final sample size of *N* = 3,375. Of these, 46.8% were male, with a mean age of 49 years (*SD* = 17.6). Most participants identified as non-Hispanic white (72.7%). In terms of education, 6.1% reported less than a high-school degree, 54.2% reported a high-school degree or some college and 39.6% reported an undergraduate degree or higher. The median annual household income was 45,000–74,999 U.S. dollars. We employed sample weights provided by the survey team to account for the complex sample design of the ANES and to adjust for demographic discrepancies from U.S. population estimates due to nonresponse.

#### Measures

##### Cognitive ability

To obtain a proxy for cognitive ability, we used the 10-item Wordsum test which is a subset of items from the Thorndike-Gallup (Thorndike, 1942; Thorndike & Gallup, 1944) test of verbal intelligence. Previous work demonstrated that verbal ability scores are closely related to more comprehensives tests of general intelligence (Miner, 1957; Wolfle, 1980; Zhu & Weiss, 2005). The Wordsum test consists of 10 multiple-choice items, each of which presents participants with one target word and five response options (one correct and four distractors). Participants are asked to identify the word whose meaning is closest to the target word (e.g., “Tell me the number of the word that comes *closest* to the meaning of the word BEAST”: 1 = *afraid*; 2 = *words*; 3 = *large*; 4 = *animal*; 5 = *separate*). A total test score was generated by averaging the number of correct answers, with higher scores indicating a higher proportion of correct answers (*M* = .68, *SD* = .24; KR-20 = .75; McDonald’s ω = .75).

##### Economic attitudes

To measure economic ideology, we selected those questions in the 2016 ANES that (a) covered participants attitudes toward economic policy issues and (b) were measured on a continuous scale. We identified eight questions that met these criteria. An exploratory factor analysis of these items revealed a single factor (eigenvalue = 3.57) explaining 45% of the total variance. All items loaded highly on this factor (≥ .57), except for one question regarding attitudes toward free trade agreements (−0.17). The item was removed from the final scale and is examined separately in the Online Supplementary Materials. The questions were recoded to range from 0 to 1 and averaged to form an economic attitudes composite (*M* = .46, *SD* = .20; Cronbach’s α = .81; McDonald’s ω = .85). Higher scores on this scale reflect more conservative views on economic issues.

##### Socioeconomic status

Socioeconomic status was assessed by educational attainment (0 = *less than high school*; 0.25 = *high school degree*; 0.50 = *some college/associate degree*; 0.75 = *undergraduate degree*; and 1 = *graduate degree; M* = .49, *SD* = .29) and self-reported annual household income, recoded to quantiles (0 = *less than $22,500*; 0.25 = *$22,500–$44,999*; 0.50 = *$45,000–$74,999*; 0.75 = *$75,000–$109,999*; and 1 = *$110,000 or more; M* = .50, *SD* = .36).

##### Politico-economic knowledge

Unfortunately, the 2016 ANES questionnaire contains only two genuinely economic knowledge questions: about the current national unemployment rate and the minimum wage in participant’s state. Since economic and political knowledge are highly correlated (Delli Carpini & Keeter, 1996), we decided to build an index from both knowledge domains to increase the reliability of the measure. The index consists of 11 factual knowledge questions across a wide range of American politics. All answers were coded as either correct or incorrect and averaged to construct an index of politico-economic knowledge, such that higher scores indicate a higher proportion of correct answers (*M* = .53, *SD* = .23; KR-20 = 0.72; McDonald’s ω = .73).

##### Epistemic needs

Following Johnston et al. (2017), we assessed epistemic needs using the Authoritarian Child-Rearing Values (ACRV) scale. Even though the ACRV is considered a measure of authoritarianism rather than a direct measure of epistemic needs, past research argued that authoritarian individuals are more sensitive to threat and intolerant of uncertainty (e.g., Feldman, 2003; Jost et al., 2003). Consequently, authoritarian child-rearing values haven been used as a reasonable proxy for heightened needs for security and certainty (Johnston et al., 2017). In the ACRV scale, articipants are asked to choose among four pairs of qualities that children should learn (“independence or respect for elders,” “curiosity or good manners,” “obedience or self-reliance,” and “being considerate or well behaved”). Authoritarian answers are coded 1, neutral answers (don’t know, neither) are coded 0.50, and non-authoritarian answers are coded 0. An overall score was computed by taking the sum divided by the total number of items (*M* = .56, *SD* = .32; Cronbach’s α = .64; McDonald’s ω = .65) with higher scores indicating a heightened need for certainty.

##### Controls

The demographic controls were age, sex (1 = *male* and 0 = *female*), and self-identified race (1 = *non-Hispanic white* and 0 = *other*).

### Results and Discussion

At the bivariate level, cognitive ability was weakly positively associated with economic conservatism, *r* = .05, *t* = 2.65, *p* = .008, 95% CI = [0.01, 0.08]. The magnitude of the correlation was fairly similar to the overall effect size reported in Study 1 and fell within the 95% confidence interval of the meta-analytic effect size estimate. The full set of bivariate correlations among all study variables are available in the Supplementary Materials.

**Figure 4.**
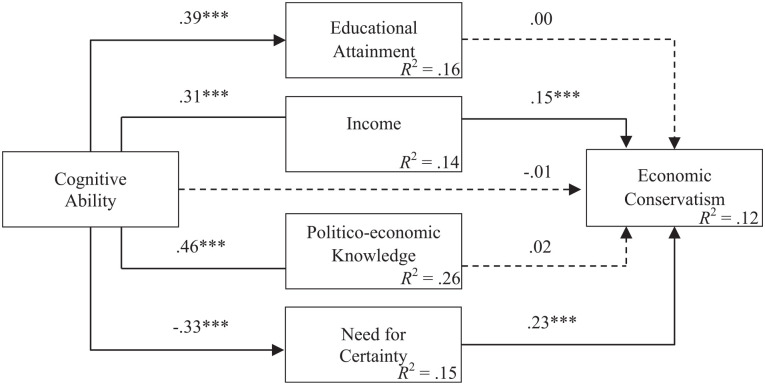
Path model showing the relationships between cognitive ability and economic conservatism as mediated by socioeconomic status, politico-economic knowledge, and epistemic needs. *Note.* Entries are standardized path coefficients for the full model. The full model includes the direct effects of demographic covariates on all endogenous variables, which for the sake of brevity are not shown. The dashed lines indicate nonsignificant paths (*p* > .05), *N* = 3,375. Weighted data. *p < .05. **p < .01. ***p < .001.

To test the hypothesized multiple mediator model, we estimated a fully saturated path model with observed variables using the maximum likelihood method.^8^ As shown in Figure 4, cognitive ability was significantly positively related to educational attainment (β = .39, *SE* = .02, *t* = 19.10, *p* < .001, 95% CI = [0.35, 0.43]), income (β = .31, *SE* = .02, *t* = 15.81, *p* < .001, 95% CI = [0.27, 0.35]), and politico-economic knowledge (β = .46, *SE* = .02, *t* = 23.25, *p* < .001, 95% CI = [0.42, 0.49]). In turn, only income was significantly linked to economic conservatism (β = .15, *SE* = .02, *t* = 6.79, *p* < .001, 95% CI = [0.11, 0.20]). As expected, cognitive ability was significantly negatively related to higher need for certainty (β = −.33, *SE* = .02, *t* = −15.85, *p* < .001, 95% CI = [−0.38, −0.29]), which in turn was positively linked to more conservative views on economic issues (β = .23, *SE* = .02, *t* = 12.15, *p* < .001, 95% CI = [0.19, 0.27]). After adjusting for demographic covariates and the mediating variables, the direct effect of cognitive ability on economic conservatism was non-significant (β = −.01, *SE* = .02, *t* = −0.29, *p* = .77, 95% CI = [−0.06, 0.04]). Overall, the model explained 12.2% of the variance in economic attitudes.

Finally, the indirect effects of cognitive ability were tested using the Monte Carlo method to construct 95% confidence intervals with 5,000 replications (Preacher & Selig, 2012). Since there are no significant paths between educational attainment or politico-economic knowledge and economic attitudes, we restrict our analysis to the effects of income and epistemic needs. The results of the mediation analysis provided significant empirical evidence for both indirect effects: First, for a positive indirect effect of cognitive ability on economic conservatism that is mediated through income (β = .05, *SE* = .01, *z* = 6.20, *p* < .001, 95% CI = [0.03, 0.06]), which is consistent with the self-interest hypothesis. Second, for a negative indirect effect of cognitive ability on economic conservatism that is mediated through need for certainty (β = −.08, *SE* = .01, *z* = 12.15, *p* < .001, 95% CI = [−0.09, −0.06]), which is consistent with the epistemic needs hypothesis.

## General Discussion

In the present research, we investigated the association of cognitive abilities with economic attitudes by synthesizing the extant empirical evidence in a meta-analysis (Study 1) and by testing hypotheses concerning possible mechanisms underlying this association that follow from different theoretical perspectives (Study 2). Our meta-analysis provided evidence for a small positive association (*r* = .07) of cognitive abilities with economic conservatism, on average. However, the effect sizes and directions of the associations were very heterogeneous. The strength of the association was moderated by several methodological features of the extant studies: It tended to be more pronounced in studies that used measures of operational rather than symbolic economic ideology (or mixed scales), in studies that used probability samples of the population rather than self-selected samples, and in studies that used Turkish, British, or Scandinavian rather than North-American samples. However, it was not moderated through the type or number of items of the cognitive ability measure that was used.

In the light of the heterogeneity of the size and sign of the association of mental abilities with economic attitudes observed in Study 1, Study 2 aimed at investigating different hypotheses that have been proposed to explain the association. Here, we found support for a mediation of a positive effect of mental abilities on economic conservatism through income. This supports the self-interest hypothesis according to which higher cognitive abilities facilitate higher social status and high-status individuals are less supportive of governmental regulations of markets, and redistributive social policies because they have more to lose from these measures than low-status individuals (Johnston, 2018). We found no support for the economic sophistication hypothesis according to which a positive association of cognitive abilities with economic conservatism is mediated through economic knowledge. However, we found support for a negative effect of cognitive abilities on economic conservatism that is mediated through need for certainty. Importantly, the fact that we found support for two hypotheses proposing countervailing effects of mental abilities on economic political attitudes through different causal mechanism offers an explanation for the weak average association and the heterogeneity of the empirical evidence we observe in Study 1.

Some points concerning our investigation of causal mechanisms in the present research need to be highlighted: First, we used correlational data to test hypotheses about causal mechanisms. The mediation analyses we conducted allow for conclusions about whether the empirical data are compatible with and support specific hypotheses about causal mechanisms. However, these analyses cannot provide strong evidence for causal effects or detect unique causal mechanisms (see Fiedler et al., 2011). Second, the theoretical perspectives and hypotheses we described and tested are far from exhaustive. The central conclusion from the pattern of results of Study 2 is that there is evidence for multiple mechanisms with sometimes countervailing effects. However, other theoretical perspectives and mechanisms than the ones we focused on might also play a role in explaining the association of mental abilities with economic political attitudes.

Third, we derived very abstract hypotheses from the theoretical perspectives we introduced to test them empirically. The formulation and empirical test of abstract hypotheses served the purpose of the current research well. However, each of the theoretical perspectives entails more precise predictions concerning the causal mechanism that links cognitive abilities to economic political attitudes. For example, the epistemic needs hypothesis holds that individuals with high epistemic needs feel attracted to economic conservatism because core elements of economic conservatism are functional for satisfying these needs. While the fact that we find evidence for a negative link between cognitive abilities and economic conservatism that is mediated through epistemic needs supports this view, it is not clear whether a functional fit indeed explains the association of epistemic needs with economic conservatism. In this respect, it has been argued that a functional link between psychological needs and political attitudes exists primarily for sociocultural but not for economic attitudes (e.g., Federico & Malka, 2018; Johnston & Wronski, 2015; Malka & Soto, 2015). From this perspective, in contexts where social and economic conservatism are communicated as a coherent package in the political discourse, individuals with high epistemic needs who are familiar with the discourse and perceive politics as personally relevant tend to endorse economic conservatism to express their identity as conservatives rather than because economic conservatism is particularly suitable to satisfy their needs (for empirical evidence, see Jedinger & Burger, 2019, 2020; Johnston et al., 2017; Malka et al., 2014).

There is much room for future research to test different theoretical assumption on specific causal links between cognitive abilities empirically. A further promising avenue for future research on the link between cognitive abilities and political attitudes lies in focusing on specific combinations of economic and sociocultural attitudes along with corresponding symbolic self-categorizations of individuals. For example, findings by Yilmaz et al. (2020) indicate that self-identified libertarians, who combine economic conservatism with liberal sociocultural views, play a crucial role in driving the association of cognitive style with economic conservatism.

Our findings should also be considered in the light of the fact that the data of the present investigation mainly encompass samples from Western, industrialized, rich, and democratic countries while cultural and national differences may have implications for the intelligence-ideology nexus. Hence, an important avenue for future research is to extend the investigation of the link of cognitive abilities with economic policy preferences to a broader set of cultural contexts.

## Supplemental Material

sj-pdf-1-psp-10.1177_01461672211046808 – Supplemental material for Do Smarter People Have More Conservative Economic Attitudes? Assessing the Relationship Between Cognitive Ability and Economic Ideology Click here for additional data file. Supplemental material, sj-pdf-1-psp-10.1177_01461672211046808 for Do Smarter People Have More Conservative Economic Attitudes? Assessing the Relationship Between Cognitive Ability and Economic Ideology by Alexander Jedinger and Axel M. Burger in Personality and Social Psychology Bulletin

## Notes

1. Note that we use the terms in the US-American sense. In a European context a conservative economic worldview is typically referred to as economic liberalism, that is, endorsement of economic freedom and support for a competitive market economy. The opposing view is typically referred to as economic leftism or state interventionism.

2. Proponents of this view acknowledge that within societies with different historical trajectories conservatism can be characterized differently: “[I]t is at least conceivable that the two core aspects of left-right ideology [. . .] are decoupled or even negatively correlated in socialist or communist regimes and that this could be due to the marshaling of system justification tendencies in support of egalitarian rather than hierarchical ideals” (Jost et al., 2009 p. 329).

3. We also included online databases that cover articles from non-WEIRD countries (SciELO, Redalyc, African Journals OnLine, CiNii, J-STAGE, China Integrated Knowledge Resources Database CNKI). We revisit this issue in the discussion section.

4. The exact search string was ALL = ([ attitudes OR economic beliefs OR economic ideology OR economic conservatism OR economic liberalism] AND [cognitive ability OR mental ability OR intelligence OR IQ OR wordsum])

5. Additional analyses indicate that the overall association persists when controlling for educational attainment as a potential confounder, *r* = 0.07, *z* = 2.86, *p* = .004, 95% CI = [0.02, 0.11], see the Supplemental Material available online.

6. While the confidence interval indicates the degree of uncertainty around the average effect size, the prediction interval signals the dispersion around the average effect by incorporating information about the between-study variance (τ^2^).

7. One could argue that the effect of ideological measurement is confounded by the number of items. Therefore, we performed a meta-regression in which the number of items was controlled for. The results showed that the positive association between cognitive skills and operational economic ideology was still substantial and significant, *b* = 0.224, *SE* = 0.099, *z* = 2.27, *p* = .023.

8. The residuals of all mediating variables were allowed to covary. The Supplementary Materials present additional results for the other covariates in the path model.
