# Supplementary material for: Do Smarter People Have More Conservative Economic Attitudes? Assessing the Relationship Between Cognitive Ability and Economic Ideology
Source: Pers Soc Psychol Bull. 2021 Sep 22;48(11):1548–65. doi: 10.1177/01461672211046808 (PMC9548663; doi:10.1177/01461672211046808)
Supplement: sj-pdf-1-psp-10.1177_01461672211046808 – Supplemental material for Do Smarter People Have More Conservative Economic Attitudes? Assessing the Relationship Between Cognitive Ability and Economic Ideology [file sj-pdf-1-psp-10.1177_01461672211046808.pdf]

Supplemental Materials

Do smarter people have more conservative economic attitudes? Assessing the relationship between cognitive ability and economic ideology

Alexander Jedinger & Axel M. Burger

GESIS – Leibniz Institute for the Social Sciences

Table of Contents

Appendix A: Additional Analyses on the Role of Education (Study 1)

Appendix B: Items and Question Wording (Study 2)

Appendix C: Additional Analyses (Study 2)

References

Appendix A: Additional Analyses on the Role of Education (Study 1)

In the meta-analysis, we have considered only bivariate associations between intelligence and economic conservatism. One possible objection would be that the relationships are confounded by the level of education. This means that cognitive abilities and economic attitudes are mutually dependent on educational attainment. Depending on the theoretical perspective, however, educational success can also be seen as an intervening mechanism rather than a confounding variable. First, there is evidence from longitudinal studies that intelligence predicts later educational success (Deary, Strand, Smith, & Fernandes, 2007; Schoon, Cheng, Gale, Batty, & Deary, 2010), but the direction of causality is highly debated (see Deary & Johnson, 2010 for a discussion). Second, various studies show that the associations are robust even when educational attainment is controlled for (Caplan & Miller, 2010; Carl, 2014, 2015; Ganzach, 2018, Study 1; Kanazawa, 2010, Study 2; Ludeke & Rasmussen, 2018, Study 2). Nevertheless, education could also be the cause of a spurious correlation between cognitive ability and economic ideology given that it is related not only to acquired human capital but also to political socialization in educational institutions, where ideas and values are transmitted that affect individuals' economic views (e.g., Hainmueller & Hiscox, 2006).

To determine the pure effect of intelligence, we recalculated the correlations using large-scale representative data to which we had access (ANES 2012, ANES 2016, GSS) and used the partial correlation coefficient, adjusted for education, between cognitive skills and economic ideology as a measure of effect size (n = 10,343). Controlling for educational attainment, the overall effect was positive and significant, indicating that more intelligent individuals have more conservative economic attitudes, r = 0.07, z = 2.86, p = 0.004, 95% CI [0.02, 0.11]. The variation in effect sizes was still substantial, τ^2 (effect size variance) = 0.001, τ (effect size standard deviation) = 0.04, $Q(2)$ = 11.09, p = .004, I^2 = 79.7%. As indicated by

the 95% PI, predictions range from a strong negative to a strong positive association [-0.44, 0.54].

Appendix B: Items and Question Wording (Study 2)

Cognitive Ability (Wordsum)

The exact wording of the Wordsum test is confidential.

- WORDSUM set B
- WORDSUM set D
- WORDSUM set E
- WORDSUM set F
- WORDSUM set G
- WORDSUM set H
- WORDSUM set J
- WORDSUM set K
- WORDSUM set L
- WORDSUM set O

Response coding: Correct or incorrect

Economic Attitudes

Services and spending tradeoff

Some people think the government should provide fewer services even in areas such as health and education in order to reduce spending. Suppose these people are at one end of a scale, at point 1. Other people feel it is important for the government to provide many more services even if it means an increase in spending. Suppose these people are at the other end, at point 7. And, of course, some other people have opinions somewhere in between, at points 2, 3, 4, 5 or 6. Where would you place yourself on this scale, or haven't you thought much about this?

Response scale: 1: Govt should provide many fewer services, 2, 3, 4, 5, 6, 7: Govt should provide many more services, 99: Haven't thought much about this (DO NOT PROBE)

Government or private health insurance tradeoff

There is much concern about the rapid rise in medical and hospital costs. Some people feel there should be a government insurance plan which would cover all medical and hospital expenses for everyone. Suppose these people are at one end of a scale, at point 1. Others feel that all medical expenses should be paid by individuals through private insurance plans like Blue Cross or other company paid plans. Suppose these people are at the other end, at point 7. And, of course, some other people have opinions somewhere in between, at points 2, 3, 4, 5, or 6. Where would you place yourself on this scale, or haven't you thought much about this?

Response scale: 1: Govt insurance plan, 2, 3, 4, 5, 6, 7: Private insurance plan, 99: Haven't thought much about this (DO NOT PROBE)

Guaranteed jobs and living standard tradeoff

Some people feel the government in Washington should see to it that every person has a job and a good standard of living. Suppose these people are at one end of a scale, at point 1. Others think the government should just let each person get ahead on their own. Suppose these people are at the other end, at point 7. And, of course, some other people have opinions somewhere in between, at points 2, 3, 4, 5, or 6.

Response scale: 1: Govt should see to jobs and standard of living, 2, 3, 4, 5, 6, 7: Govt should let each person get ahead on own, 99: Haven't thought much about this (DO NOT PROBE)

Government spending for health care

Do you favor an increase, decrease, or no change in government spending to help people pay for health insurance when they can't pay for it all themselves? Increase, Decrease, No change

IF R SAYS GOVT HELP FOR PEOPLE TO PAY FOR HEALTH CARE SHOULD INCREASE: Should it increase a great deal, a moderate amount, or a little?

IF R SAYS GOVT HELP FOR PEOPLE TO PAY FOR HEALTH CARE SHOULD DECREASE: Should it decrease a great deal, a moderate amount, or a little?

Free Trade Agreements

Do you favor, oppose, or neither favor nor oppose the U.S. making free trade agreements with other countries? Favor, oppose, neither favor nor oppose

IF R FAVORS U.S FREE TRADE AGREEMENTS WITH OTHER COUNTRIES: How strongly do you favor it? A great deal, moderately, or a little?

IF R OPPOSEs U.S FREE TRADE AGREEMENTS WITH OTHER COUNTRIES: How strongly do you oppose it? A great deal, moderately, or a little?

Government should reduce differences in income levels

Please say to what extend you agree or disagree with the following statement: 'The government should take measures to reduce differences in income levels'. Do you agree strongly, agree somewhat, neither agree nor disagree, disagree somewhat, or disagree strongly?

Government regulation of banks

In your opinion, when it comes to regulating the activities of banks, should the government be doing more, less, or the same as it is now? More, less, the same

IF R SAYS GOVERNMENT SHOULD BE DOING MORE TO REGULATE BANKS: How much more should the government be doing to regulate banks, a great deal, a moderate amount, or a little?

IF R SAYS GOVERNMENT SHOULD BE DOING LESS TO REGULATE BANKS:

How much less should the government be doing to regulate banks, a great deal, a moderate amount, or a little?

Government regulation of business

How much government regulation of business is good for society? A great deal, a lot, a moderate amount, a little, or none at all?

Socioeconomic Status

Education

What is the highest level of school you have completed or the highest degree you have received? (recoded to five categories): Less than high school, high school degree, some college/associate degree, BA degree, graduate degree

Annual Household Income

The next question is about [the total income of all the members of your family living here / your total income] in 2015, before taxes. This figure should include income from all sources, including salaries, wages, pensions, Social Security, dividends, interest, and all other income. What was [the total income in 2015 of all your family members living here / your total income in 2015]? TYPE THE NUMBER. YOUR BEST GUESS IS FINE (recoded into quantiles): Below $22,500; $22,500-$44,999; $45,000-$74,999; $75,000-$109,999; $110,000 or more.

Socioeconomic Knowledge

1. For how many years is a United States Senator elected – that is, how many years are there in one full term of office for a U.S. Senator? [open]

2. On which of the following does the U.S. federal government currently spend the least? Foreign aid, Medicare, National defense, Social Security
3. Do you happen to know which party currently has the most members in the U.S. House of Representatives in Washington? Democrats, Republicans
4. Do you happen to know which party currently has the most members in the U.S. Senate? Democrats, Republicans
5. Now we have a set of questions concerning various public figures. We want to see how much information about them gets out to the public from television, newspapers and the like. The first name is: Joe Biden What job or political office does he now hold? [open]
6. Paul Ryan. What job or political office does he now hold? [open]
7. Angela Merkel. What job or political office does she now hold? [open]
8. Vladimir Putin What job or political office does he now hold? [open]
9. John Roberts. What job or political office does he now hold? [open]
10. What was the current unemployment rate in the United States as of November 4 2017? 2.9%, 4.9%, 6.9%, 4.8%
11. What is the minimum wage in [sample state]? $__ per hour

Need for Certainty

Although there are a number of qualities that people feel that children should have, every person thinks that some are more important than others. I am going to read you pairs of desirable qualities. Please tell me which one you think is more important for a child to have:

1. Independence or respect for elders, both [VOL], neither [VOL]
2. Curiosity or good manners, both [VOL], neither [VOL]
3. Obedience or self□reliance, both [VOL], neither [VOL]

4. Being considerate or well behaved, both [VOL], neither [VOL]

Demographics and Controls

Age: in years

Gender: What is your gender? Male, female, other

Race: I am going to read you a list of five race categories. Please choose one or more races that you consider yourself to be: - white, - black or African-American, - American Indian or Alaska Native, - Asian, or - Native Hawaiian or other Pacific Islander? PROBE FOR RACE IF R SAYS HISPANIC OR A HISPANIC ORIGIN ENTER ALL THAT APPLY

- White, non-Hispanic
- Black, non-Hispanic
- Asian, native Hawaiian or other Pacific Islander, non-Hispanic
- Native American or Alaska Native, non-Hispanic 10 17 27
- Hispanic,
- Other non-Hispanic incl. multiple races

Appendix C: Additional Analyses (Study 2)

Table B-1. Bivariate Correlations among Main Study Variables with 95% Confidence Intervals

	1	2	3	4	5
1. Cognitive Ability					
2. Economic Conservatism	0.05<br>[0.01, 0.08]**				
3. Educational Attainment	0.39<br>[0.36, 0.42]***	0.03<br>[-0.01, 0.06]			
4. Income	0.34<br>[0.31, 0.37]***	0.16<br>[0.13, 0.20]***	0.41<br>[0.39, 0.44]***		
5. Politico-economic Knowledge	0.49<br>[0.46, 0.52]***	0.06<br>[0.03, 0.10]***	0.36<br>[0.33, 0.39]***	0.35<br>[0.32, 0.38]***	
6. Need for Certainty	-0.34<br>[-0.37, -0.31]***	0.15<br>[0.12, 0.18]***	-0.33<br>[-0.36, -0.30]***	-0.23<br>[-0.26, -0.19]***	-0.26<br>[-0.29, -0.23]***

Note. All continuous variables ranged from 0 to 1. $N = 3,375$. Weighted data.

Table B-2. Path Model Showing Relationships between Cognitive Ability and Economic Conservatism as Mediated by Socioeconomic Status, Politico-economic Knowledge, and Epistemic Needs

	Economic Conservatism		
Direct Effects	*β*	*SE*	95% CI
Cognitive Ability	-0.01	0.02	[-0.06, 0.04]
Educational Attainment	0.00	0.02	[-0.05, 0.05]
Income	0.15***	0.02	[0.11, 0.20]
Politico-economic Knowledge	0.02	0.02	[-0.02, 0.07]
Need for Certainty	0.23***	0.02	[0.19, 0.27]
Age	0.05*	0.02	[0.00, 0.09]
Male	0.07**	0.02	[0.02, 0.11]
White	0.22***	0.02	[0.18, 0.26]
Indirect Effects of Cognitive Ability			
via Educational Attainment	0.00	0.01	[-0.02, 0.02]
via Income	0.05***	0.01	[0.03, 0.06]
via Politico-economic Knowledge	0.01	0.01	[-0.01, 0.03]
via Need for Certainty	-0.08	0.01	[-0.09, -0.06]
R^2	12.2%		

Note: The entries are standardized path coefficients, standard errors, and 95% confidence intervals. All continuous variables ranged from 0 to 1, $N = 3{,}375$. Weighted data.
* $p < .05$, ** $p < .01$, *** $p < .001$.

Note that our factor analysis suggests that support for free trade has to be considered separate from other elements of economic conservatism. The bivariate correlation with cognitive ability was considerably stronger than the association of the economic attitude composite, $r = .12$, $t = 6.68$, $p < 0.001$, 95% CI [0.08, 0.15]. We repeated the path analysis with support for free trade as the dependent variable (see Table B-3). Education was significantly positively linked to free-trade attitudes ($\beta = .13$, $SE = .03$, $t = 5.29$, $p < .001$, 95% CI [0.08, 0.19]), while need for certainty were negatively associated with support for free trade ($\beta = -.12$, $SE = .02$, $t = -6.07$, $p < .001$, 95% CI [-0.16, -0.08]). The mediation analysis revealed a positive indirect effect of cognitive ability that was mediated via education ($\beta = .05$, $SE = .01$, $z = 5.07$, $p < .001$, 95% CI [0.03, 0.07]) as well as via epistemic needs ($\beta = .04$, $SE = .01$, $z = 5.65$, $p < .001$, 95% CI [0.03, 0.05]).

Table B-3. Path Model Showing Relationships between Cognitive Ability and Trade Attitudes as Mediated by Socioeconomic Status, Politico-economic Knowledge, and Epistemic Needs

	Opposition to Free Trade Agreements		
Direct Effects	*β*	*SE*	95% CI
Cognitive Ability	0.02	0.03	[-0.03, 0.08]
Educational Attainment	0.13***	0.03	[0.08, 0.19]
Income	0.01	0.02	[-0.04, 0.06]
Politico-economic Knowledge	0.01	0.03	[-0.05, 0.06]
Need for Certainty	-0.12***	0.02	[-0.16, -0.08]
Age	0.05*	0.02	[0.01, 0.10]
Male	0.03	0.02	[-0.01, 0.07]
White	-0.09***	0.02	[-0.13, -0.04]
Indirect Effects of Cognitive Ability			
via Educational Attainment	0.05***	0.01	[0.03, 0.07]
via Income	0.00	0.01	[-0.01, 0.02]
via Politico-economic Knowledge	0.00	0.01	[-0.02, 0.03]
via Need for Certainty	0.04***	0.01	[0.03, 0.05]
R^2	5.4%		

Note: The entries are standardized path coefficients, standard errors, and 95% confidence intervals. All continuous variables ranged from 0 to 1, $N = 3,335$. Weighted data.
* $p < .05$, ** $p < .01$, *** $p < .001$.

References

Caplan, B., & Miller, S. C. (2010). Intelligence makes people think like economists: Evidence from the general social survey. *Intelligence*, *38*(6), 636–647. doi: 10.1016/j.intell.2010.09.005

Carl, N. (2014). Verbal intelligence is correlated with socially and economically liberal beliefs. *Intelligence*, *44*, 142–148. doi: 10.1016/j.intell.2014.03.005

Carl, N. (2015). Cognitive ability and political beliefs in the United States. *Personality and Individual Differences*, *83*, 245–248. doi: 10.1016/j.paid.2015.04.029

Deary, I. J., & Johnson, W. (2010). Intelligence and education: Causal perceptions drive analytic processes and therefore conclusions. *International Journal of Epidemiology*, *39*(5), 1362–1369. doi: 10.1093/ije/dyq072

Deary, I. J., Strand, S., Smith, P., & Fernandes, C. (2007). Intelligence and educational achievement. *Intelligence*, *35*(1), 13–21. doi: 10.1016/j.intell.2006.02.001

Ganzach, Y. (2018). Intelligence and the rationality of political preferences. *Intelligence*, *69*, 59–70. doi: 10.1016/j.intell.2018.05.002

Hainmueller, J., & Hiscox, M. J. (2006). Learning to Love Globalization: Education and Individual Attitudes Toward International Trade. *International Organization*, *60*(2), 469–498. Cambridge Core. doi: 10.1017/S0020818306060140

Kanazawa, S. (2010). Why Liberals and Atheists Are More Intelligent. *Social Psychology Quarterly*, *73*(1), 33–57. doi: 10.1177/0190272510361602

Ludeke, S. G., & Rasmussen, S. H. R. (2018). Different political systems suppress or facilitate the impact of intelligence on how you vote: A comparison of the US and Denmark. *Intelligence*, *70*, 1–6. doi: 10.1016/j.intell.2018.06.003

Schoon, I., Cheng, H., Gale, C. R., Batty, G. D., & Deary, I. J. (2010). Social status, cognitive ability, and educational attainment as predictors of liberal social attitudes and political trust. *Intelligence*, *38*(1), 144–150. doi: 10.1016/j.intell.2009.09.005
